# Potential influence of decision time on punishment behavior and its evaluation

**DOI:** 10.3389/fpsyg.2022.794953

**Published:** 2022-08-22

**Authors:** Kaede Maeda, Yuka Kumai, Hirofumi Hashimoto

**Affiliations:** ^1^Department of Psychology, College of Contemporary Psychology, Rikkyo University, Niiza, Japan; ^2^Graduate School of Letters, Yasuda Women’s University, Hiroshima, Japan; ^3^Graduate School of Literature and Human Sciences, Osaka Metropolitan University, Osaka, Japan

**Keywords:** punishment, time pressure, evaluation, social dilemma, second-party punishment, third-party punishment

## Abstract

Previous studies on whether punishers are rewarded by reputational gains have yielded conflicting results. Some studies have argued that punitive behaviors potentially result in a positive evaluation, while others have found the opposite. This study aims to clarify the conditions that lead to the positive evaluation of costly punishment. Study 1 utilized one-round and repeated public goods game (PGG) situations and manipulated decision time for participants’ punitive behavior toward the non-cooperative person in the situation. We also asked participants to report their impression evaluations of punitive behavior toward non-cooperative people. Moreover, utilizing the second- and third-party punishment games, Study 2 manipulated the decision time of participants’ punitive behavior toward the self-interested person and asked them to evaluate the punitive behavior. The results showed that those who punished intuitively were not likely to be evaluated positively. However, punishers were rewarded when the decision to punish was made after deliberation or made by those who were not direct victims. These findings extend previous research on the evaluation of punitive behavior and reveal that deliberative punishment is evaluated positively occasionally.

## Introduction

Punishing free riders in a social dilemma has been considered the key to understanding large-scale human cooperation (Yamagishi, 1986, 1988; Fehr and Gächter, 2000). However, it is still unclear whether evaluations of punishers benefit their reputation. A possible reason as to why researchers are particularly intrigued by the evaluations that punishment behavior induces is to analyze whether costly punishment leads to reputational gains and the adaptiveness of the punishment (Barclay, 2006; Kurzban et al., 2007). Although theoretical models assume that reputational gains allow punishment to evolve (Panchanathan and Boyd, 2004), experimental studies present conflicting evidence. Punishers are more likely to be seen as trustworthy by others and be chosen as partners to play future games (Barclay, 2006; Nelissen, 2008). Horita (2010) also showed that punishers were trusted more than non-punishers, although they were chosen less frequently than non-punishers to receive rewards. In contrast, Kiyonari and Barclay (2008) demonstrated that evaluations of punishers were not improved in a public goods game (PGG). To systematically discuss these seemingly conflicting results regarding the evaluation of punishers, more empirical studies are needed to clarify the conditions under which punishers are evaluated positively (or negatively). In this paper, through two studies, we aim to clarify the conditions that lead to positive evaluation of costly punishment by distinguishing whether such punishment behaviors are based on intuition or deliberation.

Specifically, we focused on the following aspects. First, we examined the differences between intuitive and deliberative decision-making. Recent theoretical and empirical studies based on the dual-process theory (Evans, 2008; Kahneman, 2011; Evans and Stanovich, 2013) have pointed to the possibility that decisions may differ depending on whether they are based on intuition or deliberation (see Hallsson et al., 2018; Capraro, 2019; for a review). For example, Rand and Nowak (2013) have suggested that intuitive decision-making may promote reciprocal cooperative behavior. As cooperative behavior is potentially based on an intuitive response, punitive behavior against unfairness may also be based on intuition. There is some empirical evidence that the punitive behavior against unfairness exhibited in ultimatum games is based on intuitive judgments (e.g., Cappelletti et al., 2008; Wang et al., 2011). Even in situations such as PGGs, an experiment by Mischkowski et al. (2018) showed decreased punishment behavior over time. These results are in accordance with the dual-process theory in that punishment behavior is driven by an intuitive or emotional (e.g., anger or anger-related negative emotion) response to unfairness, which is then suppressed by deliberation. Given these findings and the dual-process model, we assume that the length of time allowed for participants’ decision-making affects their punitive behaviors and potentially impacts the impression evaluation of punishers. Second, Barclay (2006) states that the evaluation of punishers in an economic game depends on either repeated interactions or only one-round of interaction. According to this argument, we assume that the game type (one-round or repeated PGG situation) affects the evaluation of punitive behavior because reputation is inconsequential in a one-round game. Third, we also focused on whether or not the punishers are the direct victims in the situation; this study uses the term “partyness” to describe that the punisher is a direct victim. Raihani and Bshary (2015) concluded that punishment by direct victims tends to be perceived as an act of retaliation and, therefore, may be feared by others (see also, Kriss et al., 2016; Stüber, 2020). In contrast, third-party punishment enforcers may be considered socially desirable individuals who aim for group-beneficial norm compliance and cooperation. Thus, the context of the punisher’s partyness may influence punitive behavior and its evaluation.

The present study is divided into two parts. Study 1 incorporates the potential effect of game type (one-round or repeated PGG situation) and decision time (intuition vs. deliberation) as an independent variable and examines its effects on punishment behavior and its evaluation. Specifically, we utilize one-round and repeated PGG situations and assess the potential influence of decision time on punishment behavior and its evaluation. Study 2 utilizes the second-party punishment game (SPPG)^1^ and third-party punishment game (TPPG; Fehr and Gächter, 2002; Fehr and Fischbacher, 2004) to manipulate whether or not the punishers are the direct victims and examine the potential influence of decision time and partyness (SPPG vs. TPPG) on punishment behavior and its evaluation. These two studies consider seemingly identical punishment behavior by dividing it into intuition- and deliberation-based punishment and argue the adaptive value of punishment behavior by analyzing the impression evaluation of these two types, thereby extending existing research on the potential reputational gains of punitive behavior. To the best of our knowledge, there are no studies that analyze the impression evaluation of these two types of punishment. We believe that our study can help clarify previously inconsistent evaluations of punishment behavior, depending on whether punishment is based on intuitive or deliberative decision-making.

## Materials and methods

### Study 1: Potential effect of time pressure on the evaluation of punishers in public goods game

#### Participants

Ninety-one Japanese female undergraduates (mean age = 18.85 years, *SD* = 0.73) participated in this study. The participants were recruited from the attendees of a lecture on the introduction to social research. Forty-four participants were randomly assigned to the one-round PGG condition and 47 to the repeated PGG condition. After the lecture, participants were informed that their decision to participate was voluntary, and they were free to withdraw their consent at any point in this study. All the students who attended the lecture agreed to participate. In order to create a reasonable distance between individual participants and to guarantee the anonymity of their decision-making, the participants were moved to a larger classroom for each condition, after which the experiment was started. In this study, we used monetary rewards to incentivize participants; the instructions emphasized that 15% of the participants would be given the amount determined by their actual decision-making in the one-round or repeated PGG experiment through a prepaid card, called “QUO card,” which can be used for payment at affiliated stores.

#### Procedure

Study 1 was conducted in a classroom setting. First, the experimenter distributed the instruction sheet to the participants. The general rules of PGG were explained in detail on the screen and instruction sheet. Participants were divided into groups of four comprising students who did not know each other. They were all given JPY 800 and asked how much they wanted to contribute to their group. Participants were told that the total amount of money contributed to the group would be doubled by an experimenter and divided equally among group members. After the experimenter confirmed that all participants understood these rules, each participant was given a decision sheet in an envelope and asked to decide the contribution amount. Subsequently, participants assigned to the one-round PGG condition were informed: “this experiment is a one-time event.” Participants assigned to the repeated PGG condition were informed: “this experiment will be repeated several times in the future with the same group.^2^ “

One week later, the same participants gathered in each classroom. The experimenter explained that the PGG experiment with the punishment stage was a continuation of the previous week’s experiment and let the participants know the results of their group members’ decision-making. Regardless of the contribution, each participant was provided the same information through the feedback sheet. The information, handwritten on this feedback sheet, was fake and implied that there were selfish participants in their group. The stated result was that (a) the total amount contributed by the four participants to the group was JPY 1,200; (b) the doubled amount, JPY 2,400, would be divided equally (each participant would receive JPY 600); and (c) one group member did not contribute to the group and kept the entire JPY 800, and therefore, this person would receive JPY 1,400.^3^

The procedure for the subsequent experiments is as follows: (1) punishment decision-making task within 5 s (intuition condition); (2) evaluation task of intuitive and deliberative (non-) punishers, respectively; and (3) punishment decision-making task without a time limit (deliberation condition). This order of events was determined with the intention of shortening the overall time of the experiment; the manipulation of decision-making time (i.e., intuition and deliberation conditions) was a within-subjects design. After reading the feedback sheet, participants were asked to decide and note down how much money they would use as punishment against the selfish person within 5 s (i.e., the intuition condition).^4^ The efficiency of the punishment was three times the amount the participant paid for the punishment. The participants made their decisions by ticking one of the five possible options: “Pay JPY 0 and deduct JPY 0 from the person,” “Pay JPY 100 and deduct JPY 300 from the person,” “Pay JPY 200 and deduct JPY 600 from the person,” “Pay JPY 300 and deduct JPY 900 from the person,” and “Pay JPY 400 and deduct JPY 1,200 from the person.” Thereafter, in this situation, we asked the participants to evaluate the punishers (i.e., those who paid JPY 400 so that the selfish person would lose JPY 1,200) and the non-punishers (i.e., those who paid JPY 0 so that the selfish person would lose JPY 0) by choosing from nine options, ranging from –4 (a very bad impression) to 4 (a very good impression). Here, we distinguished between those who punished intuitively and those who did so deliberately. More specifically, we presented the person “who decided to deduct immediately” and the person “who decided to deduct after careful consideration.” When the participants had completed their evaluation, they were asked again, without a time limit, to make their decisions about punishment after careful deliberation (i.e., the deliberation condition). After the experiment and data collection was completed, participants received a debriefing: here, we informed the participants that regardless of their actual decision-making in the PGG, we gave false feedback to create a situation where each participant was informed that there was only one selfish person (the person to be punished) in their group.

#### Results and discussion of study 1

**FIGURE 1:**
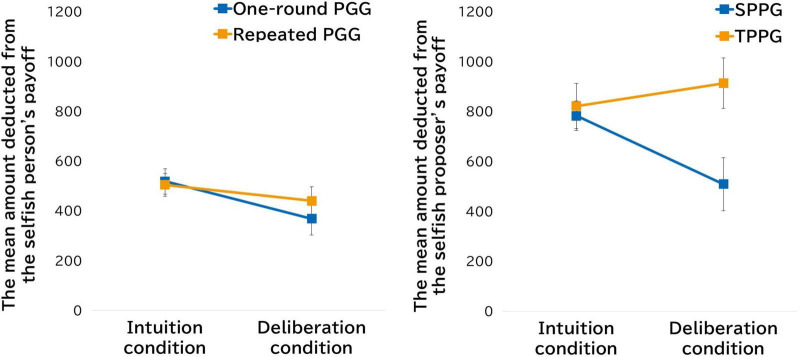
Conditional differences in mean amount spent for punishment in Study 1 and Study 2.

To examine whether the mean contribution amount differed between the conditions, we conducted a *t*-test. We found no difference between the one-round and repeated PGG conditions [*t* (88) = 1.55, *p* = 0.12, *d* = 0.32]. The mean amount deducted from the selfish person’s payoff by participants in the one-round PGG condition was JPY 518.18 in the intuition condition and JPY 368.18 in the deliberation condition. In the repeated PGG condition, the mean amount was JPY 504.26 in the intuition condition and JPY 440.43 in the deliberation condition. We conducted a 2 (game type: one-round and repeated) × 2 (decision time: intuition and deliberation) mixed-factor ANOVA for the mean amount deducted from the selfish person’s payoff. The results were statistically significant for the main effect of decision time [*F* (1,89) = 6.63, *p* = 0.01, η*_*p*_*^2^ = 0.07], although the main effect of game type [*F* (1,89) = 0.20, *p* = 0.66, η*_*p*_*^2^ = 0.002] and the interaction effect of game type and decision time [*F* (1,89) = 1.08, *p* = 0.30, η*_*p*_*^2^ = 0.01] were not significant (Figure 1). We performed an additional multiple comparison analysis to clarify the main effect of decision time, and found a significant difference in the mean amount deducted by the participants in the one-round PGG [*t* (89) = 2.51, *p* = 0.01, *d* = 0.54]. These results suggest that time pressure significantly increased the amount spent on punishment only in the one-round PGG.

**FIGURE 2:**
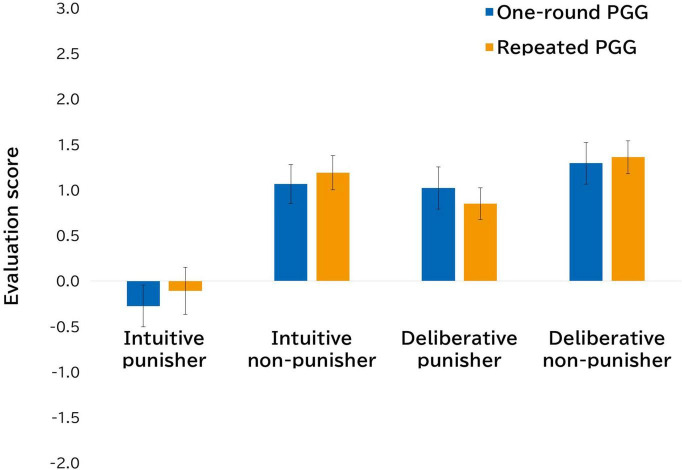
Conditional differences in mean evaluation scores of intuitive (non-) punisher and deliberative (non-) punisher (Study 1).

The results of impression evaluation of (non-) punishers also had interesting implications (Figure 2). We conducted a 2 (game type: one-round and repeated) × 2 (decision time: intuition and deliberation) × 2 (punisher: punisher or non-punisher) ANOVA for evaluation scores. The results revealed a main effect of decision time [*F* (1,89) = 43.55, *p* < 0.001, η*_*p*_*^2^ = 0.33] and punisher [*F* (1,89) = 17.95, *p* < 0.001, η*_*p*_*^2^ = 0.17], and an interaction effect of decision time × punisher [*F* (1,89) = 19.09, *p* < 0.001, η*_*p*_*^2^ = 0.18]. As shown in Figure 2, punishment based on deliberate decision-making was more likely to be evaluated positively than that based on intuitive decision-making. There was no significant effect of game type [*F* (1,89) = 0.07, *p* = 0.79, η*_*p*_*^2^ = 0.001] or related interaction effects of game type.^5^

### Study 2: Potential effect of time pressure on the evaluation of punishers in second-party punishment game and third-party punishment game

#### Participants

In Study 2, 46 Japanese female undergraduates (mean age = 19.83 years, *SD* = 0.71) participated. Participants were recruited from a lecture on cultural psychology. Twenty-three participants were randomly assigned to the SPPG condition and 23 to the TPPG condition. The experiment was conducted in a classroom setting and offered monetary rewards to incentivize participants; 15% of the participants would be given the money based on their actual decision-making through a prepaid (QUO) card.

#### Procedure

##### Instructions for the second-party punishment game condition

Using the first instruction sheet, the experimenter explained the general rules of the dictator game: (1) participants were randomly paired to play the game and assigned as either the proposer or the recipient; (2) the proposer, given JPY 1,200 by the experimenter, freely decided on a share between JPY 0 and JPY 1,200 for the recipient. After the explanation of the general rules in the first instruction sheet, the participants were informed that they had been assigned the role of recipient and given the second instruction sheet, which was distributed individually in envelopes; (3) in this instruction sheet, participants were informed that the recipients would be given JPY 400 by the experimenter; (4) the recipient could deduct an amount from the proposer’s payoff by paying any amount above JPY 400; and (5) the amount to be deducted would be three times the amount paid by the recipient. After confirming that the participants understood the rules of this game, they were given a feedback sheet (which was, like Study 1, fake) and were informed that the proposer decided to keep the JPY 1,200 for themselves.

##### Instructions for the third-party punishment game condition

As with the SPPG condition, during the first set of instructions, the general rules of the experiment were explained: (1) participants were randomly divided into groups of three and assigned as proposer, recipient, and third-party; (2) the proposer, given JPY 1,200 by the experimenter, freely decided on a share between JPY 0 and JPY 1,200 for the recipient. After the explanation of the general rules in the first instruction sheet, the participants were informed that they were assigned the role of the third-party and given the second instruction sheet, which was distributed individually in envelopes; (3) this instruction sheet informed the participants that the third-parties would be given JPY 400 by the experimenter; (4) the third-party could deduct an amount from the proposer’s payoff by paying any amount from JPY 400; and (5) the amount to be deducted was three times the amount paid by the third party. After confirming that the participants understood the rules of this game, they were given a (fake) feedback sheet and informed that the proposer had decided to keep the JPY 1,200 for themselves.

##### Intuitive and deliberative punishment

After being informed of the proposer’s decision, the recipients in the SPPG condition and third-parties in the TPPG condition were asked to note down their decision on how much money they would use to punish the selfish proposer within 5 s (intuition condition); participants made their decisions by ticking one of five possible options: “Pay JPY 0 and deduct JPY 0 from the person,” “Pay JPY 100 and deduct JPY 300 from the person,” “Pay JPY 200 and deduct JPY 600 from the person,” “Pay JPY 300 and deduct JPY 900 from the person,” and “Pay JPY 400 and deduct JPY 1,200 from the person.” Furthermore, like in Study 1, we asked the participants to report their evaluations of those who punished (i.e., those who paid JPY 400 so that the selfish proposer would lose JPY 1,200) and those who did not punish (i.e., those who paid JPY 0 so that the selfish proposer would lose JPY 0) in this situation. Study 2 also distinguished between intuitive and deliberate punishment, asking the participants to rate each type by choosing from nine options from –4 (a very bad impression) to 4 (a very good impression). In addition, like Study 1, the participants were asked again, without a time limit, to make their decisions about punishment after careful deliberation (i.e., the deliberation condition); manipulation of the decision-making time was a within-participant design. As Study 1, participants received a debriefing after the experiment and data collection was finished.

#### Results and discussion of study 2

**FIGURE 3:**
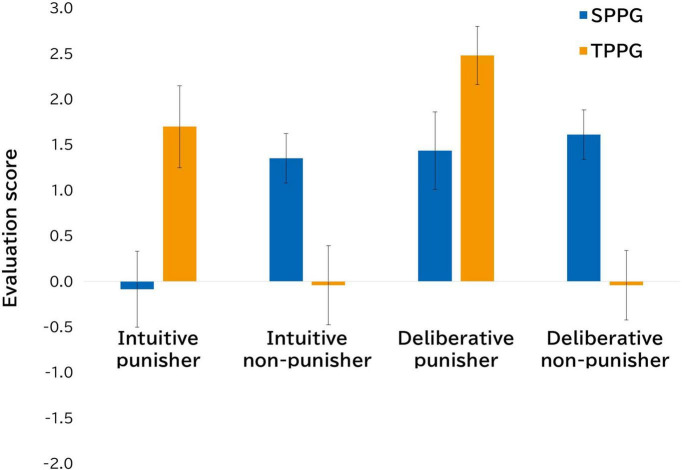
Conditional differences in mean evaluation scores of intuitive (non-) punisher and deliberative (non-) punisher (Study 2).

The mean amount deducted from the proposer’s payoff for participants playing the SPPG was JPY 782.61 in the intuition condition and JPY 508.70 in the deliberation condition. In the TPPG, the mean amount deducted was JPY 821.74 in the intuition condition and JPY 913.04 in the deliberation condition (Figure 1). We conducted a 2 (game type: SPPG and TPPG) × 2 (decision time: intuition and deliberation) mixed-factor ANOVA for the mean amount deducted from the proposer’s payoff. The results showed a main effect of game type [*F* (1,44) = 4.96, *p* = 0.03, η*_*p*_*^2^ = 0.10] and an interaction effect of game type × decision time [*F* (1,44) = 5.04, *p* = 0.03, η*_*p*_*^2^ = 0.10], although the main effect of game type [*F* (1,44) = 1.26, *p* = 0.27, η*_*p*_*^2^ = 0.03] was not significant. We performed an additional multiple comparison analysis and found significant differences in the mean amount for participants playing the SPPG [*t* (44) = 2.38, *p* = 0.02, *d* = 0.84], suggesting that the amount deducted from the proposer’s payoff in the SPPG decreases when deliberation is employed, compared with when intuitive judgment is employed. Furthermore, like Study 1, we conducted a 2 (game type: SPPG and TPPG) × 2 (decision time: intuition and deliberation) × 2 (punisher: punisher or non-punisher) ANOVA for the evaluation scores. The results revealed a main effect of decision time [*F* (1,44) = 9.31, *p* < 0.01, η*_*p*_*^2^ = 0.17], an interaction effect of game type × punisher [*F* (1,44) = 17.43, *p* < 0.01, η*_*p*_*^2^ = 0.28], and an interaction effect of decision time × punisher [*F* (1,44) = 7.48, *p* < 0.01, η*_*p*_*^2^ = 0.15].^6^ The interaction of decision time × punisher replicated the findings of Study 1, which demonstrated generally positive evaluations of punishment based on deliberation (Figure 3). In addition, the game type × punisher interaction effect suggested that the punisher in the TPPG is evaluated more positively, which indicates that punishers may be rewarded when the decision to punish is made by those who are not direct victims (i.e., TPPG).

## Discussion

Punishing those who violate group-beneficial norms plays an essential role in promoting human cooperation (Fehr and Gächter, 2002). However, it is currently ambiguous whether engaging in the costly punishment can lead to a favorable evaluation. In this study, we assumed that it is important to clarify that punishment behaviors that appear the same on the surface can be evaluated differently depending on whether they are based on intuition or deliberation. Thus, we focused on the potential influence of decision time in the evaluation of punishers in economic games and examined the role of decision time in punishment behavior and its evaluation. Our results demonstrated that (1) intuitive punishers were not likely to be positively evaluated; (2) punishers may be rewarded only when the decision to punish was made after careful deliberation or when the decision was made by those who were not the direct victims (i.e., TPPG). Therefore, based on these results, one possible reason why punishment is evaluated positively (or negatively) involves whether the evaluator perceives the costly punisher to be deliberate (or intuitive). Such an evaluation-axis may well sort out the inconsistencies in the evaluation of punishers in previous studies. However, our results suggesting that the punisher can expect to obtain a good reputation after the deliberation need careful interpretation. If this is the case, then deliberation should promote punishment behavior. However, our results did not consistently demonstrate that deliberation promotes punishment behavior; in fact, we observed the opposite tendencies. One possible reason for this is that participants who made deliberation-based decision may have punished less because they believed that a punisher would be more likely to be perceived by others as intuitive (in fact, punishers tend to decide intuitively; Cappelletti et al., 2008; Wang et al., 2011; Mischkowski et al., 2018), and therefore, it was better not to punish non-cooperators to avoid a bad reputation. Needless to say, we cannot strongly argue that this interpretation is correct; further study is needed. Furthermore, although their applicability needs to be thoroughly examined in the future, these findings contribute to research on the evaluation of punishment by distinguishing whether such punishment behaviors are based on intuition or deliberation.

The findings of this study are consistent with Raihani and Bshary (2015). In their framework, the punishment signal can be either cooperative or competitive. Whether the signal is interpreted as cooperative or competitive depends on the observer’s estimates of the punisher’s motivation, and the punisher’s reputation is determined by the estimation of their motivation. Furthermore, observers might fear competitive punishers; therefore, they are not evaluated favorably. In contrast, cooperative punishers are regarded more favorably and receive a positive evaluation; they are more likely to be chosen as partners (Mifune et al., 2020; Tateishi et al., 2021). It should be also noted that, in the present study, neither the punishers nor non-punishers received negative evaluations in Study 1 and 2,^7^ especially when such punishment was the result of the punishers’ deliberation. However, in the case of spontaneous punishment based on intuition, evaluations depend on whether punitive behavior is regarded as competitive or cooperative. Although the reasons why deliberative punishment is more likely to receive favorable evaluations need to be examined in more detail, these results are of interest and have the potential to reformulate Raihani and Bshary’s framework in the light of the dual-process theory.

Several issues remain to be addressed. First, while we conducted studies focusing only on whether punishment is given or not, prior research (e.g., FeldmanHall et al., 2014) suggests that people prefer compensation (e.g., compensating the victim for bad things to his or her benefit) to punishment (e.g., subtracting from the benefits of the person who did the bad thing). Furthermore, although the current study focused only on the effects of decision time, other studies (e.g., Gordon and Lea, 2016) regarding evaluations of (non-) punishers indicate that people with a high status may receive positive evaluations when they punish. As for punishment efficiency, a previous study (Nelissen, 2008) reported that the greater the cost of punishment, the more positive the evaluation. It is necessary to consider the possibility that punishment efficiency affects punitive behavior and its evaluation. It is noteworthy that our participants were asked to make a judgment about punishment behavior under time pressure (intuition condition) and then make the same judgment again with no time limit (deliberation condition). This procedure has much in common with the “two-response paradigm” that has been developed to distinguish and compare intuitive and deliberative judgments (e.g., Thompson et al., 2011; Bago and De Neys, 2019; Hashimoto et al., 2022). Although the findings are interesting, there is a potential limitation in that the deliberation condition always follows the intuitive one. If we assume that participants’ understanding is deepened through repeated decision-making, then the later deliberation condition may lead to more “rational” decision-making, regardless of time pressure. It should also be noted that the present study asked participants to evaluate the punishers prior to the deliberation condition. Therefore, future studies are needed to determine whether these results can be replicated using a between-participant design or counterbalancing the conditions. Another limitation is that this study utilized the Likert scale to evaluate impressions of punishers. Future studies need to conduct more precise impression evaluation measurements, such as using specific adjectives (trustworthy, etc.) in addition to reporting only good or bad impressions, or whether to choose a person as a partner in an experimental game under conditions of monetary reward incentives. Additionally, it should be noted that we used deception in our experiment. It is undeniable that this manipulation may have influenced the participants’ evaluations, and therefore, future studies without deception are necessary. Finally, it is also potentially problematic that the generalizability of our results is limited to young Japanese female students. Prior research demonstrates that the Japanese tend to avoid negative evaluations in social contexts (e.g., Hashimoto and Yamagishi, 2013, 2016) and adopt strategies to appease people who meet the expectations of others by default (Yamagishi et al., 2008, 2012; Hashimoto and Yamagishi, 2015); these tendencies are more pronounced in the Japanese youth (Hashimoto, 2021). Thus, Japanese youth, who tend to focus on avoiding negative evaluations, may be less likely to consider the potential positive effects of punishment. Therefore, it is necessary to conduct systematic cross-cultural research on Japanese adults in general.

## Data availability statement

The raw data supporting the conclusions of this article will be made available by the authors, without undue reservation.

## Ethics statement

The studies involving human participants were reviewed and approved by the Yasuda Women’s University. The patients/participants provided their written informed consent to participate in this study.

## Author contributions

KM, YK, and HH contributed to the study design, analyzed data, and wrote the whole part of the manuscript. KM and YK conducted data collection. All authors contributed to the article and approved the submitted version.

## Footnotes

1 To compare the conditions of whether the punishers are the direct victims in the games or not, we utilized a slightly modified dictator game with the punishment option (i.e., SPPG). The primary difference between the SPPG and TPPG lies in the partyness; that is, whether participants were direct victims or not (third-parties).

2 Participants in the repeated PGG condition were informed that the second period of the PGG would be conducted 1 week later to intimate that the game would be repeated. However, they did not actually repeat the game.

3 Since each participant’s contribution to the PGG was not the same, even if the same information was provided, strict control of the experiment was difficult. It is undeniable that participants’ cooperative tendencies may have influenced punishment behavior and the evaluation of punishment. However, this is unlikely to be a major issue because this potential problem did not occur in Study 2 which utilized a one-time game and still found similar tendencies.

4 Following Maeda and Hashimoto (2020)’s experimental procedure, we limited decision-making in the intuition condition to 5 s. By giving detailed instructions on when to turn the questionnaire over and close it, the experimenter controlled the decision-making time (5 s) for participants to answer to the decision-making sheet. It should be noted here that the classroom setting could not be strictly limited to a 5-s period. However, there is no doubt that the participants in this intuition condition were made sensitive to the time pressure.

5 There were no significant interaction effects of game type × decision time [*F* (1,89) = 0.97, *p* = 0.33, η*_*p*_*^2^ = 0.011], game type × punisher [*F* (1,89) = 0.06, *p* = 0.81, η*_*p*_*^2^ = 0.001], and game type × decision time × punisher [*F* (1,89) = 0.44, *p* = 0.51, η*_*p*_*^2^ = 0.005].

6 There were no significant main effects of game type [*F* (1,44) = 0.04, *p* = 0.85, η*_*p*_*^2^ = 0.001] and punisher [*F* (1,44) = 3.56, *p* = 0.07, η*_*p*_*^2^ = 0.075]. There were also no significant interaction effects for game type × decision time [*F* (1,44) = 1.41, *p* = 0.24, η*_*p*_*^2^ = 0.031] and game type × decision time × punisher [*F* (1,44) = 0.41, *p* = 0.53, η*_*p*_*^2^ = 0.009].

7 One-sample *t*-tests of the evaluation scores in Study 1 and Study 2, which were conducted as an additional exploratory analysis, demonstrated that none of the results (*t*s ≤ 1.19 in Study 1; *t*s ≤ 0.21 in Study 2) were significantly below the theoretical median (0).

## Abbreviations

PGG: public goods game
SPPG: second-party punishment game
TPPG: third-party punishment game.
